# Examination of the Association between Insufficient Sleep and Cardiovascular Disease and Diabetes by Race/Ethnicity

**DOI:** 10.1155/2011/789358

**Published:** 2011-06-27

**Authors:** Abhishek Vishnu, Anoop Shankar, Sita Kalidindi

**Affiliations:** ^1^Department of Community Medicine, West Virginia University School of Medicine, P.O. Box 9190, Morgantown, WV 26506-9190, USA; ^2^Department of Statistics, West Virginia University, Morgantown, WV 26506, USA

## Abstract

*Background*. We examined the association between insufficient rest/sleep and cardiovascular disease or diabetes mellitus separately among non-Hispanic whites, non-Hispanic blacks, Hispanic Americans, and other races in a contemporary sample of US adults. *Methods*. Multiethnic, nationally representative, cross-sectional survey (2008 BRFSS) participants who were >20 years of age (*n* = 369, 217; 50% women). Self-reported insufficient rest/sleep in the previous month was categorized into: zero, 1–13, 14–29, and all 30 days. Outcomes were: (1) any CVD, (2) coronary artery disease (CHD), (3) stroke, and (4) diabetes mellitus. *Results.* Insufficient rest/sleep was found to be positively associated with (1) any CVD, (2) CHD, and (3) stroke among all race-ethnicities. In contrast, insufficient rest/sleep was positively associated with diabetes mellitus in all race-ethnicities except non-Hispanic blacks. The odds ratio of diabetes association with insufficient rest/sleep for all 30 days was 1.37 (1.26–1.48) among non-Hispanic whites, 1.11 (0.90–1.36) among non-Hispanic blacks, 1.88 (1.46–2.42) among Hispanic Americans, and 1.48 (1.10–2.00) among other race/ethnicities. *Conclusion*. In a multiethnic sample of US adults, perceived insufficient rest/sleep was associated with CVD, among all race-ethnicities. However, the association between insufficient rest/sleep and diabetes mellitus was present among all race-ethnicities except non-Hispanic blacks.

## 1. Introduction

Sleep loss, long-term sleep deprivation, and perceived insufficient rest/sleep are common in modern society [1]. To study the sleep pattern of the U.S. population, the Centers for Disease Control and Prevention included a new survey question on insufficient rest/sleep in the 2008 Behavioral Risk Factor Surveillance System (BRFSS) core questionnaire. A recent report based on this survey estimated that 70% of US population has at least some degree of insufficient rest/sleep and that there were variations in perceived insufficient rest/sleep by race/ethnicity [2]. 

Insufficient rest/sleep may be related to various social and economic factors such as low education, unemployment, job stress, urban environment, late-night socializing, noisy neighborhoods, and a disturbed home environment [3]. Earlier studies have reported variations in the presence of such stressors among different race-ethnicities [3–7]. 

Recent studies have reported an association between insufficient sleep and cardiovascular disease [8–10]. However, few studies have examined the association between insufficient rest/sleep and outcomes such as coronary heart disease, stroke, and diabetes. Therefore, we examined the association between perceived insufficient rest/sleep, cardiovascular disease and diabetes mellitus among different race-ethnicities after adjusting for main confounding factors in the 2008 BRFSS survey, which is a large, multiethnic sample of US adults. 

## 2. Methods

### 2.1. Study Population

The BRFSS is a federally funded nationally representative survey of the civilian, noninstitutionalized, adult population aged 18 years or older. The survey is designed and conducted annually by the CDC in collaboration with the state health departments to monitor health-related behaviors and risk factors in the US population. The survey selects state-specific probability samples of households using a multistage cluster design to produce a nationally representative sample. The BRFSS uses random-digit dialing within blocks of telephone numbers to identify a probability sample of households with telephones in each state. In each household, one adult is randomly identified and interviewed. All 50 states, in addition to the District of Columbia and the three US territories participated in the 2008 BRFSS. Detailed description of the BRFSS survey sample selection and study methodology are available online [11]. In 2008, the median response rate was 75.0% [12]. 

To examine the association between insufficient rest/sleep and cardiovascular disease, diabetes mellitus, and obesity, out of the 414,509 BRFSS participants, we excluded subjects who were aged <20 years, pregnant, or who had missing information on variables included in the current analysis, including insufficient rest/sleep, physician-diagnosed cardiovascular disease or diabetes, body mass index (BMI), smoking, education, or physical activity. This resulted in 369,217 adults (50% of whom were women) with complete covariate data for the current analysis. Out of these, 42,213 subjects had any cardiovascular disease, 33,423 had coronary heart disease, 14,290 had stroke, and 41,291 had diabetes mellitus.

### 2.2. Main Outcomes of Interest: Cardiovascular Disease and Diabetes Mellitus

The 2008 BRFSS core module asked questions about physician-diagnosed history of coronary heart disease and stroke. A positive response to these questions was taken as evidence of the presence of coronary heart disease or stroke, respectively. In the current study, we also defined “any cardiovascular disease” as the presence of self-reported coronary heart disease or stroke. Similarly, diabetes mellitus was defined as a positive response to questions on physician-diagnosed history of diabetes mellitus.

### 2.3. Exposure Measurements

 The 2008 BRFSS survey included the question, “During the past 30 days, for about how many days have you felt you did not get enough rest/sleep?” This question was previously tested and validated in the 2006 BRFSS survey in four states (Delaware, Hawaii, New York, and Rhode Island) [1]. Data from all sites were aggregated, and the numbers of days of perceived insufficient rest/sleep were categorized into four groups as zero days, 1–13 days, 14–29 days, and 30 days.

 We are using data from the 2008 BRFSS survey conducted by the CDC. The national prevalence of insufficient rest/sleep was reported by CDC researchers in a 2009 JAMA report [2] using the same categories (zero days, 1–13 days, 14–29 days, and 30 days). Furthermore, several subsequent publications by independent researchers have used the same insufficient rest/sleep categories [13, 14]. Therefore, we are using the same insufficient rest/sleep categories used by CDC and other researchers so that our results are directly comparable to these previous reports from the same data.

 Age, gender, race/ethnicity, smoking status, alcohol intake, level of education, and physical activity were assessed using a standardized questionnaire. Race/ethnicity was divided into four main categories as Whites, Blacks or African Americans, Hispanic Americans, and others. Individuals who had not smoked >100 cigarettes in their lifetimes were classified as never smokers; those who had smoked >100 cigarettes in their lifetimes were classified as former smokers or current smokers based on their response to the question on current smoking. Heavy-alcohol drinking was defined as men who reported having more than 2 drinks per day, or women that reported having more than 1 drink per day. Education was categorized into below high school, high school, or above high school education. Employment status was categorized as employed, unemployed, retired, or unable to work. BMI was categorized into <25, 25–29, and ≥30 kg/m^2^. Subjects were classified as having no regular exercise if they reported not to be participating in any physical activities such as running, calisthenics, golf, gardening, or walking for exercise during the previous month.

### 2.4. Statistical Analysis

We examined the characteristics of the study sample by calculating the mean values of continuous variables and frequencies of categorical variables. Perceived insufficient rest/sleep was categorized into four groups as zero days, 1–13 days, 14–29 days, and 30 days. We had four outcomes of interest: (1) any cardiovascular disease, (2) coronary heart disease, (3) stroke, and (4) diabetes mellitus. We examined the association between insufficient rest/sleep and these outcomes separately by race-ethnicity. We used logistic regression models to calculate odds ratio [(OR) (95% confidence interval (CI)] of each outcome of interest associated with increasing categories of insufficient rest/sleep, taking zero days of insufficient rest/sleep as the referent category. We used two nested logistic regression models: the age and sex-adjusted model and the multivariable model, additionally controlling for education categories (<high school, high school, >high school), smoking (never, former, current), employment status (employed, unemployed, retired, unable to work), heavy drinker (no, yes), BMI categories (<25, 25–29, and ≥30 kg/m^2^) and no regular exercise (yes, no). Trends in the OR of each outcome across increasing insufficient rest/sleep category were determined by modeling these categories as an ordinal variable. Additionally, interaction between insufficient rest/sleep and race was tested by including cross-product multiplicative interaction terms in multivariable logistic regression models. Appropriate BRFSS survey weights that account for unequal probabilities of selection, oversampling, and non-response were applied for all analyses using SUDAAN (version 8.0; Research Triangle Institute, Research Triangle Park, NC, USA) and SAS (version 9.2; SAS Institute, Cary, NC, USA) software; SEs were estimated using the Taylor series linearization method.

## 3. Results

Table 1 presents the characteristics of the study population. Half of the study sample were women, approximately 17% were subjects >65 years of age, 8% were non-Hispanic blacks, while 6.5% were Hispanic Americans. Approximately, 62% of the study subjects had above high school education, 62% were employed, 19% were current smokers, 5% were heavy alcohol drinkers, and 25% reported no regular exercise. About 27% of the subjects were obese, 9% had diabetes mellitus, and 8% had any history of cardiovascular disease. Overall, 31% subjects reported zero days of insufficient rest/sleep while 11% reported all 30 days of insufficient rest/sleep in the past month. Non-Hispanic blacks were more likely than other race-ethnicities to smoke, be unemployed, have more than normal weight, have diabetes, and to report insufficient rest/sleep on all 30 days of the month.

Table 2 presents the odds ratios of CVD prevalence by insufficient rest/sleep categories in the total survey population and also stratified by different race-ethnicities. There was a positive association between insufficient rest/sleep and increased prevalence of CVD after age, sex, and after multivariable adjustment. (*P*-trend <.0001 in all race-ethnicity categories). 

**Figure 1:**
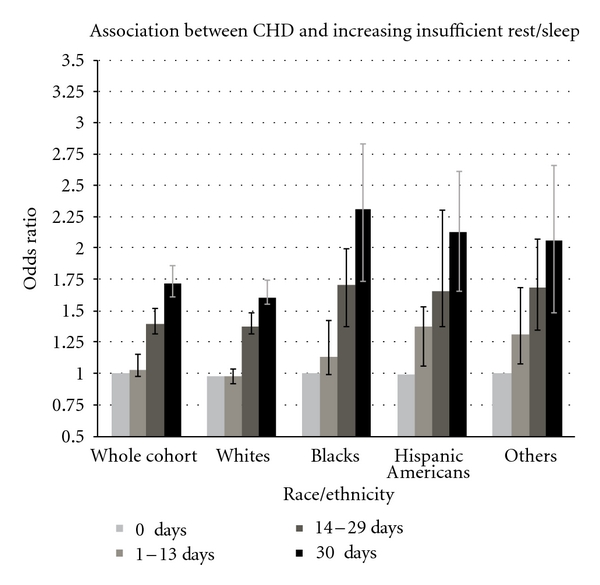
Association between insufficient rest/sleep and coronary heart disease (CHD). *X*-axis: categories of insufficient rest/sleep within the whole population and by different race-ethnicities. *Y*-axis: odds ratio of coronary heart disease prevalence (95% CI).

**Figure 2:**
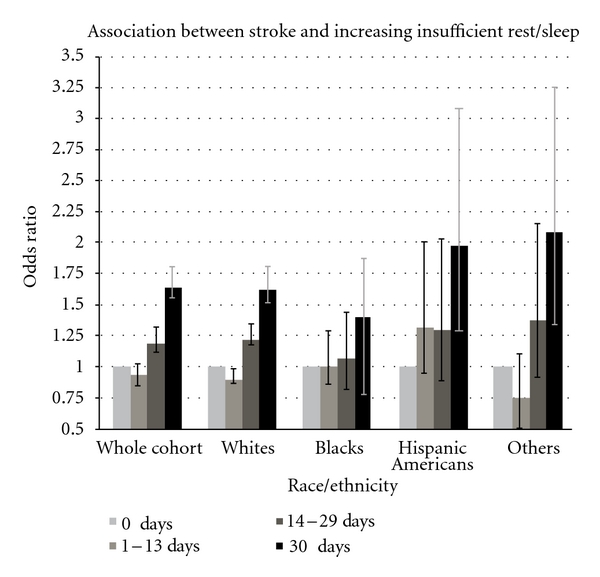
Association between insufficient rest/sleep and stroke. *X*-axis: categories of insufficient rest/sleep within the whole population and by different race-ethnicities. *Y*-axis: odds ratio of stroke prevalence (95% CI).

 Figures 1 and 2 present the association between increasing categories of insufficient rest/sleep and components of any cardiovascular disease, including coronary heart disease and stroke, respectively, in the total survey population and stratified by race-ethnicity. The positive association between insufficient rest/sleep and cardiovascular disease was consistently present in separate analysis examining coronary heart disease and stroke as the outcome of interest. The association remained positive after stratification by race-ethnicity.

Table 3 presents our finding of a positive association between insufficient rest/sleep and diabetes mellitus after age, sex, and after multivariable adjustment. After stratifying sample by race-ethnicity and applying age sex adjustment, we found a positive association among all race-ethnicities (*P* < .01 for all race-ethnicities). However, when we applied multivariable adjustment, additionally controlling for lifestyle factors, all race-ethnicities except non-Hispanic blacks had a positive association (*P*-trend = .1019 for blacks, *P*-trend <.05 for other race-ethnicities).

 When we performed formal statistical tests of interaction, we found that the *P*-interaction values were significant for all outcomes, including any CVD (*P*-interaction = .041), coronary heart disease (*P*-interaction = .032), stroke (*P*-interaction = .043), and diabetes (*P*-interaction <.001). In a supplementary analysis, we examined the association between insufficient rest/sleep and the various outcomes by considering age in categories (20–30 years, 30–40 years, 40–50 years, 50–60, years and >60 years) employing dummy coding instead of a continuous variable; the results for any CVD, coronary heart disease, stroke, and diabetes were found to be essentially similar to our main findings presented in the tables. For example, for any CVD as the outcome, compared to zero days of insufficient rest/sleep (referent), the multivariable-adjusted OR (95% CI) was 1.01 (0.96–1.06) for 1–13 days, 1.37 (1.29–1.47) for 14–29 days and 1.76 (1.64–1.89) for 30 days; *P*-trend <.0001. In a second supplementary analysis, we included quadratic terms for age and BMI in the multivariable model. Here, also, we found that the results were essentially the same. For example, for any CVD as the outcome, compared to zero days of insufficient rest/sleep (referent), the multivariable-adjusted OR (95% CI) was 1.02 (0.95–1.09) for 1–13 days, 1.36 (1.27–1.46) for 14–29 days, and 1.77 (1.63–1.92) for 30 days; *P*-trend <.0001. 

In a third supplementary analysis, we used the number of days of insufficient rest/sleep as a continuous variable to determine its effect on any CVD, coronary heart disease, stroke, and diabetes. Consistent with our main findings, we found a positive association between insufficient rest/sleep and any CVD, coronary heart disease, and stroke in all racial-ethnic groups; in contrast, for diabetes a positive association was present in all racial-ethnic groups except non-Hispanic blacks. For example, for diabetes as the outcome, the multivariable-adjusted OR (95% CI) of insufficient rest/sleep as a continuous variable was 1.18 (1.16–1.20) for the whole cohort, 1.12 (1.08–1.16) for whites, 0.98 (0.89–1.08) for blacks, 1.22 (1.17–1.27) for Hispanic Americans, and 1.19 (1.16–1.22) for other racial-ethnic groups.

## 4. Discussion

 In a large representative sample of U.S. adults, we found a positive association between insufficient rest/sleep and cardiovascular disease, including coronary heart disease and stroke, in separate analyses among all major racial-ethnic groups. The association was independent of age, sex, educational status, smoking, employment status, alcohol intake, regular exercise, and body mass index. In contrast, insufficient sleep was found to be positively associated with diabetes mellitus among all race-ethnicities except non-Hispanic blacks. Our results suggest that there are ethnic differences in the relation between insufficient sleep and chronic diseases and highlight the need to examine the relation between sleep and health by race-ethnicity.

 In the current study, the large size of the sample, the magnitude of the association between insufficient rest/sleep and various outcomes, and the persistence of such association after multivariable adjustment of confounding variables suggest that these findings are less likely to be due to chance. Earlier studies have also reported racial differences in sleep pattern [13, 15] and association of short sleep duration with cardiovascular disease [8, 9, 16, 17] and diabetes mellitus [18–21]. Findings of our current study are consistent with these reports.

 Social and economic disparities have been implicated in sleep differences among different racial-ethnic groups [3]. Residents of poor neighborhoods are more likely to report stress due to uncertain future, multiple jobs, shift work, or depression which might prevent them from falling asleep. It might also be explained by crowded living conditions, lack of privacy, and noise pollution prevalent in the inner city areas [3]. 

 Earlier studies in the US [22, 23], Germany [24], Japan [25], and Canada [18] have reported association between insufficient rest/sleep and diabetes mellitus. Non-Hispanic blacks are known to have higher prevalence of insufficient rest/sleep [1] as well as diabetes mellitus [26] than the general population.

 In the current study, we observed a positive association between insufficient rest/sleep and diabetes mellitus in all race-ethnicities after age and sex adjustment. However, when we applied multivariable adjustment, where we additionally controlled for socioeconomic and lifestyle factors, the association between sleep and diabetes mellitus among non-Hispanic blacks disappeared, but was still present in other race-ethnicities. Our results are consistent with another study by Beihl et al. that reported a similar ethnic difference in the association between insufficient sleep and diabetes [27]. However, the mechanism responsible for such an effect is not entirely clear. We believe that these relations are driven partly by underlying sleep disorders and partly by environment. Our analysis suggests that the association between insufficient rest/sleep and diabetes mellitus among non-Hispanic blacks is explained by variables included in the multivariable model such as education, smoking, BMI, and lack of physical activity. Previous reports have shown that non-Hispanic blacks may have lower educational status [3], higher unemployment [7], higher BMI [28], higher rates of current smoking [29], and lower exercise duration [29]. Many of these factors are independently associated with both insufficient rest/sleep and diabetes mellitus [30–33]. 

 As our data is derived from a cross-sectional survey, our analysis suffers from inherent limitations. We could not test the temporal association between insufficient rest/sleep and cardiovascular disease or diabetes. It is possible that the large sample size of the study may make even smaller effect sizes statistically significant. Therefore, some of the statistically significant associations between insufficient rest/sleep and CVD among different race-ethnicities reported in our study may be an effect of the large sample size and not clinically important associations. We had data for insufficient rest/sleep but not for excessive sleep which is also reported to be associated with cardiovascular diseases [9]. The outcome definitions of cardiovascular disease and diabetes were based on self-reported information which could have resulted in misclassification. We believe this misclassification is likely to be non-differential, and therefore, unlikely to underestimate the true association. Additionally, the question on insufficient rest/sleep is a relatively crude measure that combines quantity and quality of sleep into one variable. It is possible that this has low reliability and validity, and therefore, our results are potentially biased as a result of exposure misclassification. Future studies with more objective measures are required to study sleep quantity and quality separately in relation to CVD and diabetes and to confirm our findings.

 In summary, in a large representative sample of US adults, increasing categories of insufficient rest/sleep were found to be positively associated with cardiovascular disease including coronary artery disease and stroke in all race-ethnicities. Insufficient rest/sleep is also associated with diabetes mellitus in all race-ethnic groups except non-Hispanic blacks. Thus, the relation between insufficient rest/sleep and diabetes mellitus should be examined by race-ethnicity in future studies to confirm or disprove our findings from a national survey.

## Figures and Tables

**Table 1:** Baseline Characteristics of study populations (percentages ± standard errors, SE) (*n* = 369,217).

Characteristic	Overall (*n* = 369, 217)	Whites (*n* = 296, 430)	Blacks (*n* = 28, 495)	Hispanics (*n* = 24, 213)	Others (*n* = 20, 079)
**Women, %**	50.05 ± 0.17	50.72 ± 0.18	53.20 ± 0.63	47.13 ± 0.64	44.71 ± 0.79
**Age group, %**					
<25 yrs	8.54 ± 0.15	7.22 ± 0.15	9.10 ± 0.48	13.35 ± 0.59	11.18 ± 0.76
25 to 34 yrs	18.83 ± 0.16	16.55 ± 0.16	20.95 ± 0.57	27.20 ± 0.62	21.57 ± 0.72
35 to 44 yrs	20.09 ± 0.14	18.86 ± 0.14	21.60 ± 0.49	23.62 ± 0.52	23.06 ± 0.65
45 to 64 yrs	35.29 ± 0.15	37.39 ± 0.16	35.13 ± 0.55	26.69 ± 0.52	32.01 ± 0.70
>65 yrs	17.25 ± 0.09	19.98 ± 0.11	13.22 ± 0.32	9.13 ± 0.27	12.18 ± 0.42
**Educational categories, %**					
Below high school	10.18 ± 0.12	5.85 ± 0.08	11.85 ± 0.36	30.61 ± 0.61	9.23 ± 0.47
High school	27.53 ± 0.15	27.21 ± 0.15	32.98 ± 0.58	28.58 ± 0.60	20.64 ± 0.61
Above high school	62.29 ± 0.17	66.93 ± 0.16	55.17 ± 0.61	40.81 ± 0.64	70.13 ± 0.72
**Employment status**					
Employed	61.90 ± 0.16	62.04 ± 0.16	59.19 ± 0.59	62.45 ± 0.62	63.24 ± 0.76
Unemployed	5.72 ± 0.10	4.37 ± 0.08	10.53 ± 0.42	8.39 ± 0.39	7.02 ± 0.44
Retired	16.35 ± 0.09	18.86 ± 0.11	13.74 ± 0.33	7.88 ± 0.26	12.18 ± 0.43
Unable to work	5.16 ± 0.07	4.48 ± 0.07	9.54 ± 0.32	5.42 ± 0.25	5.26 ± 0.27
Others	10.86 ± 0.12	10.24 ± 0.12	7.00 ± 0.33	15.86 ± 0.48	12.30 ± 0.56
**Smoking, %**					
Current Smoker	18.74 ± 0.14	18.84 ± 0.14	21.34 ± 0.51	16.38 ± 0.51	19.00 ± 0.64
Former Smoker	25.43 ± 0.14	28.41 ± 0.15	17.66 ± 0.43	18.52 ± 0.48	20.58 ± 0.63
Never Smoker	55.83 ± 0.17	52.75 ± 0.17	60.99 ± 0.59	65.11 ± 0.62	60.42 ± 0.80
**Heavy alcohol drinker, %**					
(Men: >2 drinks/day, women: >1 drinks/day)	5.34 ± 0.08	5.77 ± 0.09	3.65 ± 0.24	4.82 ± 0.32	4.45 ± 0.34
**No regular exercise**	25.12 ± 0.15	22.65 ± 0.14	31.62 ± 0.56	32.98 ± 0.60	24.62 ± 0.70
**Body mass index categories, %**					
Normal weight (<25 kg/m^2^)	35.66 ± 0.17	37.15 ± 0.17	26.03 ± 0.55	30.24 ± 0.61	45.74 ± 0.83
Over weight (25–29 kg/m^2^)	37.02 ± 0.17	36.95 ± 0.17	36.10 ± 0.60	39.42 ± 0.64	34.05 ± 0.76
Obese (>30 kg/m^2^)	27.32 ± 0.15	25.91 ± 0.15	37.87 ± 0.59	30.34 ± 0.60	20.20 ± 0.58
**Diabetes mellitus, %**	8.91 ± 0.09	7.98 ± 0.08	13.86 ± 0.37	9.80 ± 0.34	9.40 ± 0.40
**Cardiovascular disease, %**	8.28 ± 0.08	8.70 ± 0.08	8.67 ± 0.28	6.35 ± 0.27	7.52 ± 0.36
**Perceived lack of rest/sleep categories, %**					
0 days/month	30.73 ± 0.16	29.70 ± 0.15	28.66 ± 0.53	35.79 ± 0.62	33.62 ± 0.79
1–13 days/month	41.44 ± 0.17	42.24 ± 0.17	41.10 ± 0.61	39.34 ± 0.65	38.16 ± 0.81
14–29 days/month	16.77 ± 0.13	17.33 ± 0.14	16.76 ± 0.50	14.22 ± 0.45	16.36 ± 0.56
30 days/month	11.06 ± 0.11	10.72 ± 0.11	13.48 ± 0.40	10.64 ± 0.37	11.86 ± 0.49

**Table 2:** Association between insufficient rest/sleep and any cardiovascular disease (CVD).

Categories of insufficient rest/sleep	No. at risk	No. with CVD	Age-, sex-adjusted odds ratio (95% confidence intervals)	Multivariable odds ratio (95% confidence intervals)
Whole cohort				
0 days	133265	18561	1 (referent)	1 (referent)
1–13 days	144187	12014	0.93 (0.88–0.98)	0.99 (0.94–1.05)
14–29 days	55983	5920	1.46 (1.37–1.56)	1.34 (1.25–1.43)
30 days	38709	6149	2.25 (2.10–2.40)	1.72 (1.60–1.85)
*P*-trend			<0.0001	<0.0001
Whites				
0 days	105491	15476	1 (referent)	1 (referent)
1–13 days	116983	9647	0.87 (0.82–0.91)	0.95 (0.90–1.00)
14–29 days	45077	4676	1.42 (1.33–1.52)	1.32 (1.24–1.41)
30 days	28879	4586	2.14 (1.99–2.29)	1.62 (1.51–1.74)
*P*-trend			<.0001	<.0001
Blacks				
0 days	9806	1228	1 (referent)	1 (referent)
1–13 days	10690	1029	1.00 (0.83–1.21)	1.11 (0.92–1.34)
14–29 days	4180	522	1.54 (1.22–1.93)	1.49 (1.18–1.88)
30 days	3819	626	2.28 (1.82–2.84)	2.06 (1.63–2.59)
*P*-trend			<.0001	<.0001
Hispanic Americans				
0 days	9516	801	1 (referent)	1 (referent)
1–13 days	8408	650	1.35 (1.06–1.72)	1.30 (1.02–1.65)
14–29 days	3247	319	1.70 (1.27–2.28)	1.49 (1.12–1.99)
30 days	3042	443	2.80 (2.12–3.69)	2.22 (1.69–2.90)
*P*-trend			<.0001	<.0001
Others				
0 days	7146	854	1 (referent)	1 (referent)
1–13 days	7211	583	1.18 (0.92–1.52)	1.17 (0.91–1.51)
14–29 days	3094	346	2.01 (1.47–2.74)	1.71 (1.22–2.40)
30 days	2628	427	2.81 (2.10–3.77)	2.08 (1.54–2.81)
*P*-trend			<.0001	<.0001

*Adjusted for age (years), sex (men, women), education categories (<high school, high school, and >high school), smoking (never, former, current), employment status (employed, unemployed, retired, and unable to work), heavy drinker (no, yes), body mass index categories (<25, 25–29, ≥30 kg/m^2^), no regular exercise (yes, no); *P*-interaction for insufficient rest/sleep and race-ethnicity was .041.

**Table 3:** Association between insufficient rest/sleep and diabetes mellitus.

Categories of insufficient rest/sleep	No. at risk	No. with diabetes	Age-, sex-adjusted Odds Ratio (95% confidence intervals)	Multivariable Odds Ratio (95% confidence intervals)
Whole cohort				
0 days	133265	16993	1 (referent)	1 (referent)
1–13 days	144187	12964	0.95 (0.90–1.00)	1.03 (0.97–1.08)
14–29 days	55983	6058	1.26 (1.18–1.34)	1.20 (1.12–1.28)
30 days	38709	5674	1.75 (1.63–1.87)	1.42 (1.32–1.53)
*P*-trend			<.0001	<.0001
Whites				
0 days	105491	12438	1 (referent)	1 (referent)
1–13 days	116983	9319	0.89 (0.84–0.94)	0.96 (0. 91–1.01)
14–29 days	45077	4425	1.25 (1.17–1.34)	1.15 (1.07–1.24)
30 days	28879	3897	1.75 (1.62–1.88)	1.37 (1.26–1.48)
*P*-trend			<.0001	<.0001
Blacks				
0 days	9806	2060	1 (referent)	1 (referent)
1–13 days	10690	1748	0.98 (0.85–1.14)	1.04 (0.89–1.22)
14–29 days	4180	732	1.27 (1.05–1.55)	1.24 (1.01–1.52)
30 days	3819	749	1.24 (1.03–1.50)	1.11 (0.90–1.36)
*P*-trend			0.0033	0.1019
Hispanic Americans				
0 days	9516	1291	1 (referent)	1 (referent)
1–13 days	8408	968	1.22 (1.01–1.47)	1.25 (1.03–1.51)
14–29 days	3247	476	1.58 (1.25–2.00)	1.49 (1.16–1.92)
30 days	3042	530	2.10 (1.65–2.69)	1.88 (1.46–2.42)
*P*-trend			<.0001	<.0001
Others				
0 days	7146	1029	1 (referent)	1 (referent)
1–13 days	7211	821	1.32 (1.04–1.67)	1.34 (1.05–1.72)
14-29 days	3094	372	1.32 (1.00–1.73)	1.11 (0.85–1.46)
30 days	2628	436	1.94 (1.47–2.56)	1.48 (1.10–2.00)
*P*-trend			<.0001	0.0206

*Adjusted for age (years), sex (men, women), education categories (<high school, high school, >high school), smoking (never, former, current), employment status (employed, unemployed, retired, unable to work), heavy drinker (no, yes), body mass index categories (<25, 25–29, ≥30 kg/m^2^), no regular exercise (yes, no); *P*-interaction for insufficient rest/sleep and race-ethnicity was <.001.
